# Third-Generation CALPHAD Modeling of Elemental Nb and Zr and Partial Re-Assessment of Their Binary Phase Diagram

**DOI:** 10.3390/ma17235978

**Published:** 2024-12-06

**Authors:** Gabriele Traversari, Mariano Casu, Roberto Orrù, Alberto Cincotti, Alessandro Concas, Giacomo Cao, Antonio Mario Locci

**Affiliations:** 1 Dipartimento di Ingegneria Meccanica, Chimica e dei Materiali, Università degli Studi di Cagliari, Via Marengo 2, 09123 Cagliari, Italy; gabriele.traversari@unica.it (G.T.); mariano.casu2@unica.it (M.C.); roberto.orru@unica.it (R.O.); alberto.cincotti@unica.it (A.C.); alessandro.concas@unica.it (A.C.); giacomo.cao@unica.it (G.C.); 2 Centro Interdipartimentale di Ingegneria e Scienze Ambientali (CINSA), University of Cagliari, Via San Giorgio 12, 09124 Cagliari, Italy

**Keywords:** CALPHAD, Nb, Zr, third generation, thermodynamic properties, Einstein model, two-state model, phase diagram, metastability

## Abstract

Liquid metals and metallic alloys often exist as metastable phases or can be undercooled below their equilibrium melting point. The Traditional CALPHAD (CALculation of PHAse Diagrams) approach struggles to accurately model these metastable conditions, which are important in rapid quenching techniques like additive manufacturing, and to understand glass formation or oxidation phenomena occurring in the liquid phase during nuclear and high-temperature aerospace applications. On the contrary, the third-generation CALPHAD models have the potential to accurately describe metastable phase diagrams to provide better predictions of molten phase behavior under non-equilibrium conditions. The latter approach is utilized in this study to achieve a more accurate description of the thermodynamic properties of elemental Nb and Zr, with a particular focus on their liquid phases. By incorporating available first-principles data, the representation of the liquid state is improved for both elements, capturing the peculiar behavior of the heat capacity in a wide temperature range. These improvements enable a more reliable prediction of phase stability and liquidus boundaries in the Nb-Zr system. A partial re-assessment of the Nb-Zr binary phase diagram is also conducted with refined predictions of liquidus boundaries that align well with experimental data.

## 1. Introduction

Refractory metals, along with their alloys and compounds, play a crucial role in technologies and industries requiring materials that can withstand extreme conditions. These metals have exceptionally high melting points, excellent strength at elevated temperatures, and resistance to wear, corrosion, and thermal shock, so that they are very suitable, often indispensable, for applications in aerospace, nuclear energy, and several manufacturing sectors. Their ability to maintain structural integrity under harsh environments makes them ideal, for instance, in jet engines, space exploration, and high-temperature reactors. In addition, refractory metals can help drive technological advances in fields that demand both durability and performance [1,2].

The application and property domains of refractory metal-based materials have recently been extended by the concept of high entropy alloys (HEAs) and ceramics (HECs) [3,4,5,6]. These advanced materials show a variety of complex, multi-element crystal structures that can maintain their stability in unconventional temperature and pressure ranges due to the entropic stabilization arising from the randomness of multiple constituent elements. The resulting unique microstructures often lead to exceptional properties like improved toughness, hardness, and thermal stability. As a subset of these materials, refractory high-entropy alloys (RHEAs) are composed of five or more elements, typically in near-equal concentrations, where each element is a refractory metal [7,8,9].

Designing high-entropy materials, including alloys and compounds, presents several significant challenges due to their complexity and the unconventional nature of their composition. Navigating the vast compositional space that characterizes this class of materials to identify the most promising ones is a major challenge, as small changes in composition can drastically alter phase stability, microstructure, and eventually properties. Accurately predicting the stable phases in high-entropy systems is difficult because, albeit entropic stabilization of these materials can promote unexpected solid solution phases, the multi-component composition may also lead to the formation of complex intermetallics or phase separation, so that the design process is further complicated.

Addressing these challenges with the aim of tailoring the composition of high-entropy materials for specific applications requires advanced computational techniques. Latter ones may represent a fast and inexpensive method for an accurate selection of families of materials to be experimentally investigated. In this regard, the well-established CALPHAD approach may be used to explore the thermodynamic stability of high-entropy alloys and compounds [10,11,12]. In this way, elemental composition can be identified as a priori so that the formation of specific phases during the synthesis and processing stages can be favored or avoided depending on the desired properties.

It is worth mentioning that the thermodynamic properties of metastable phases (e.g., liquid elements under their melting points, specific crystal structures outside the temperature ranges where they are experimentally stable, etc.) are essential in the CALPHAD method. However, experimental measurement of thermodynamic properties of the metastable phase is extremely challenging, if not impossible. Therefore, advanced thermodynamic models closer to material physics are needed to improve the predictive capability of the CALPHAD approach for the description of metastable states. Along these lines, the development of the third-generation CALPHAD models can make the thermodynamic description of solid and liquid elemental transition metals more accurate in a wider range of temperatures by including their metastability conditions. Specifically, this approach has been applied to Au [13], Co [14,15], Cr [16,17], Cu [18], Fe [19,20], Mn [21], Mo [22], Nb [22], Ni [16], Ta [17,22], Ti [20], V [17,20], W [22,23], and Zn [24].

To the best of the authors’ knowledge, a complete third-generation-based description of Nb and Zr has not been reported in the literature. Previous thermodynamic models of Nb were limited to the solid phase [25,26]. Jiang et al. described the thermodynamic properties of solid BCC A2 and liquid Nb by segmenting the Gibbs free energy description in three temperature intervals and assumed a constant value of the liquid heat capacity for temperatures higher than the melting point [27]. A more accurate description of liquid Nb was provided by Zhang et al., but the modeling of FCC A1 and HCP A3 crystal structures was not considered in their study [22]. In the case of Zr, previous attempts were proposed only for the solid phases [25,28,29], while a third-generation-based approach for the liquid phase has not been reported so far in the literature.

This work presents a third-generation thermodynamic description of elemental Nb and Zr, and the binary Nb-Zr phase diagram is partially re-assessed. The description of the heat capacity of solid Nb and Zr takes into account different contributions, e.g., lattice vibrations, electronic excitations, and anharmonic vibrations. The Einstein model is used to describe the harmonic contribution of the lattice vibrations. Moreover, liquid Nb and Zr heat capacities are considered using a modeling approach known as the two-state model. Nb and Zr in their gas state are also described to check the possible re-stabilization of solid phases at high temperatures. Model parameters are fitted to experimental data, while model prediction capability is evaluated through comparison to empirical outcomes not used for the fitting process. The paper is organized as follows: experimental and assessed data available in the literature are reviewed in Section 2; the adopted thermodynamic model is illustrated in Section 3; and results are presented and discussed in Section 4. Finally, the main conclusions are summarized in Section 5.

## 2. Literature Review of Experimental and Assessed Data

### 2.1. Pure Nb

The experimental information of elemental Nb has been thoroughly reviewed by [30]. Heat capacity data for the solid BCC A2 and gas phase, selected therein, are adopted in this work. The original experimental data are instead used to validate the proposed thermodynamic description through the full prediction of BCC A2 solid phase enthalpy and to assess the liquid–amorphous phase. Specifically, enthalpy data for BCC A2 Nb are from [31,32,33,34]. Heat capacity and enthalpy data for the liquid phase are taken from [35] and [32,33,34,36,37], respectively.

### 2.2. Pure Zr

Arblaster has collected and reviewed experimental information on elemental Zr [38]. The reported data for the solid BCC A2, HCP A3, and gas phase were adopted in this work. Empirical data of heat capacity taken from [39,40] and enthalpy from [36,41] were used for the assessment of the liquid phase. Enthalpy information from [41,42,43] was selected for validation through the full prediction of the thermodynamic description of BCC A2 and HCP A3 solid phases.

### 2.3. The Nb-Zr System

To our knowledge, the only two assessments available for this system are from [44,45]. Using the same unary endmembers and the same experimental findings of [44,45], the new interaction parameters were calculated, taking into account data resulting from DFT calculations, obtaining comparable results. Both assessments did not show the formation of any stable compound and described the thermodynamic properties of elements according to the second generation of CALPHAD databases [46]. In the present work, Fernández Guillermet’s assessment [44] is taken into account for the comparison with the binary phase diagram resulting from the proposed third-generation-based thermodynamic description.

## 3. Thermodynamic Model

### 3.1. Pure Elements

In the 1995 Ringberg workshop, a physically based approach was suggested to model the heat capacity of pure elements in both solid and liquid phases to better describe this property in a wider temperature range [47,48]. Accordingly, the molar heat capacity of a non-magnetic crystalline or amorphous solid phase ϕ can be described through the following equation [14]:

(1) $$C_{p}^{\phi }=3R{\left(\frac{\theta_{E}^{\phi }}{T}\right)}^{2}\frac{\exp\left(\frac{\theta_{E}^{\phi }}{T}\right)}{{\left[\exp\left(\frac{\theta_{E}^{\phi }}{T}\right)-1\right]}^{2}}+a^{\phi }T+b^{\phi }T^{\nu^{\phi }},$$

which is based on the Einstein model of solids [49]. The first term is the contribution from the harmonic lattice vibrations and $\theta_{E}^{\phi }$ is the Einstein temperature of solid phase ϕ. The second term consists of the linear contributions from the electronic excitation and low order anharmonic lattice vibrations. The third term represents the contribution of high-order anharmonic lattice vibrations, where ν is an integer that can be assigned in the range of 2 to 4, depending on the shape of the experimentally observed temperature dependence of the heat capacity of phase ϕ. The last two terms of Equation (1) contain also the correction from the volume constant heat capacity, $C_{V},$ to the pressure constant one, $C_{p}$, correlated to the variation of the molar volume as a function of temperature.

Based on the available experimental data for the selected solid phase ϕ, the value of ν can be set, and the coefficients a and b can be subsequently obtained by fitting. The corresponding Einstein temperature $\theta_{E}$ can be either estimated by independent methods or fitted together with the other coefficients appearing in Equation (1) (see next). A detailed description of the adopted optimization procedure will be discussed in a dedicated section (see next).

Enthalpy at zero K is given by the relationship $H_{0}=E_{0}+\frac{3}{2}R\theta_{E}$, where $E_{0}$ is the cohesive energy at 0 K, while $\frac{3}{2}R\theta_{E}$ gives the vibrational contribution known as the zero point energy (ZPE) [19]. In general, both terms can be obtained by means of various approaches (e.g., DFT calculations) or as fitted parameters. Regarding the entropy at 0 K, it is well known that according to the third law of thermodynamics, it must be $S_{0}=0$. Integration of Equation (1), thus, yields the following expression for the other molar thermodynamic properties related to $C_{p}$ [13,50]:

(2) $$H^{\phi }=E_{0}^{\phi }+\frac{3}{2}R\theta_{E}^{\phi }\frac{\left[\exp\left(\frac{\theta_{E}^{\phi }}{T}\right)+1\right]}{\left[\exp\left(\frac{\theta_{E}^{\phi }}{T}\right)-1\right]}+\frac{a^{\phi }}{2}T^{2}+\frac{b^{\phi }}{\nu^{\phi }+1}T^{\nu^{\phi }+1},$$

(3) $$S^{\phi }=3R\left\{\frac{\frac{\theta_{E}^{\phi }}{T}}{\left[\exp\left(\frac{\theta_{E}^{\phi }}{T}\right)-1\right]}-\ln\left[1-\exp\left(-\frac{\theta_{E}^{\phi }}{T}\right)\right]\right\}+a^{\phi }T+\frac{b^{\phi }}{\nu^{\phi }}T^{\nu^{\phi }},$$

(4) $$G^{\phi }=E_{0}^{\phi }+\frac{3}{2}R\theta_{E}^{\phi }+3RT\ln\left[1-\exp\left(-\frac{\theta_{E}^{\phi }}{T}\right)\right]-\frac{a^{\phi }}{2}T^{2}-\frac{b^{\phi }}{\nu^{\phi }\left(\nu^{\phi }+1\right)}T^{\nu^{\phi }+1}.$$

At least in principle, the model described above can be applied regardless of the specific crystal structures of solid phase ϕ and its thermodynamic stability.

Typically, the Gibbs free energy for the different phases is expressed by referring to the value of enthalpy of the standard element reference (SER) (i.e., stable phase of the element at $T_{SER}$ = 298.15 K and $p_{SER}$ = 1 × 10^5^ Pa), $H_{SER}^{\alpha }$ [46]. In this work, the superscript α indicates the stable phase of each element at the standard state, while the subscript SER refers to the standard temperature and pressure conditions. Accordingly, $H_{SER}^{\alpha }$ can be expressed as follows:

(5) $$H_{SER}^{\alpha }=E_{0}^{\alpha }+\frac{3}{2}R\theta_{E}^{\alpha }\frac{\left[\exp\left(\frac{\theta_{E}^{\alpha }}{T_{SER}}\right)+1\right]}{\left[\exp\left(\frac{\theta_{E}^{\alpha }}{T_{SER}}\right)-1\right]}+\frac{a^{\alpha }}{2}T_{SER}^{2}+\frac{b^{\alpha }}{\nu^{\alpha }+1}T_{SER}^{\nu^{\alpha }+1}.$$

By defining Gϕ0=Gϕ−HSERα and combining Equations (4) and (5), the following expression can be obtained:

(6) Gϕ0=E0ϕ−E0α+ZSERα+GEINθEϕ,T−PϕT,

where

(7) $$Z_{SER}^{\alpha }=-\frac{3}{2}R\theta_{E}^{\alpha }\frac{\left[\exp\left(\frac{\theta_{E}^{\alpha }}{T_{SER}}\right)+1\right]}{\left[\exp\left(\frac{\theta_{E}^{\alpha }}{T_{SER}}\right)-1\right]}+P^{\alpha }\left(T_{SER}\right),$$

(8) $$GEIN\left(\theta_{E}^{\phi },T\right)=\frac{3}{2}R\theta_{E}^{\phi }+3RTln\left[1-\exp\left(-\frac{\theta_{E}^{\phi }}{T}\right)\right],$$

(9) $$P^{\phi }\left(T\right)=-\frac{a^{\phi }}{2}T^{2}-\frac{b^{\phi }}{\nu^{\phi }\left(\nu^{\phi }+1\right)}T^{\nu^{\phi }+1}.$$

The two-state model is adopted in this work to describe the liquid phase [51,52]. It is assumed that atoms in this phase can be either in a liquid-like state (translational state) or in an amorphous-like state (vibrational state). Consequently, this model describes a liquid–amorphous phase from the amorphous-like state that predominates at low temperatures to the liquid-like state prevailing at high temperatures. The internal variable, ξ, is defined to represent the fraction of liquid-like atoms, such that ξ=0 and ξ=1 indicate the ideal amorphous phase and the pure liquid-like state, respectively. The heat capacity of the liquid–amorphous phase can be derived as follows [23,53]:

(10) $$C_{p}^{liq-am\;}=C_{p}^{am\;}-C\xi_{eq}+\left(A^{liq}-C^{liq}T\right)\frac{d\xi_{eq}}{dT},$$

where $\xi_{eq}$ is the equilibrium fraction of liquid-like atoms, which is dependent on temperature. The first term in Equation (10) is the heat capacity of the ideal amorphous phase $\left(\xi_{eq}=0\right)$ given by Equation (1). The last two terms are the liquid-like atoms’ contribution to the two-state phase’s heat capacity. The reader should refer to Equation (11) for the meaning of coefficients $A^{liq}$ and $C^{liq}$.

To determine the equilibrium fraction of liquid-like atoms, $\xi_{eq}$, appearing into Equation (10), the difference in the Gibbs free energy between the liquid and the amorphous state is introduced as ${∆G}^{liq-am}=G^{liq}-G^{am}$. Temperature dependence of ${∆G}^{liq-am}$ is given by the following relation [19]:

(11) $${∆G}^{liq-am}=A^{liq}-B^{liq}T+C^{liq}T\ln T,$$

where $A^{liq}$, $B^{liq}$, and $C^{liq}$ are adjustable parameters to be estimated or optimized by fitting to the experimental data (see next).

The Gibbs energy of the liquid–amorphous phase can be finally formulated as follows:

(12) $$G^{liq-am}=G^{am}+\xi {∆G}^{liq-am}+RT\left[\left(1-\xi \right)\ln\left(1-\xi \right)+\xi \ln\left(\xi \right)\right],$$

where the first term, $G^{am}$, represents the molar Gibbs energy of the ideal amorphous phase given by Equation (4).

Under equilibrium conditions, the fraction of the liquid-like atoms has a value that minimizes the Gibbs energy of the two-state phase. The corresponding expression for the equilibrium fraction of the liquid-like atoms, $\xi_{eq}$, can be thus obtained under the condition $\frac{dG^{liq-am}}{d\xi }=0$; it yields:

(13) $$\xi_{eq}=\frac{1}{1+\exp\left(\frac{{∆G}^{liq-am}}{RT}\right)},$$

while the temperature derivative of $\xi_{eq}$ is given by

(14) $$\frac{d\xi_{eq}}{dT}=\frac{\left(A^{liq}-C^{liq}T\right)}{RT^{2}}\left(\xi_{eq}-\xi_{eq}^{2}\right).$$

Equation (13) has a sigmoid shape that starts from 0, at 0 K, and reaches asymptotically one only for temperature values infinitely high. It is worth noting that, depending on the analytical expression of ${∆G}^{liq-am}$, a different pseudo-asymptotic value may be reached at high temperatures [53].

As already illustrated for the solid phases, the following expressions of molar enthalpy, entropy, and Gibbs free energy of the liquid–amorphous phase are provided when integrating Equation (10):

(15) $$H^{liq-am}=H^{am}+\xi_{eq}\left(A^{liq}-C^{liq}T\right),$$

(16) $$S^{liq-am}=S^{am}-\xi_{eq}\left[B^{liq}+C^{liq}\left(1+\ln T\right)\right]-G2ST\left({∆G}^{liq-am}\right),$$

(17) $$G^{liq-am}=G^{am}+G2ST\left({∆G}^{liq-am}\right),$$

where $H^{am}$ and $S^{am}$ are calculated through Equations (2) and (3), respectively, and

(18) $$G2ST\left({∆G}^{liq-am}\right)=-RT\ln\left[1+\exp\left(-\frac{{∆G}^{liq-am}}{RT}\right)\right].$$

Taking into account Equations (6)–(9) we have also that:

(19) Gliq−am0=E0am−E0α+ZSERα+GEINθEam,T−PamT+G2ST∆Gliq−am.

Finally, the molar Gibbs free energy of pure elements in the gas phase can be expressed by the following polynomial function:

(20) Ggas0=a0gas+a1gasTlnT+∑i=210aigasTi.

### 3.2. Solution Phases

The molar Gibbs free energy of solid, liquid, and gaseous solutions can be obtained as follows:

(21) Gmixϕ0=GmixϕREF+GmixϕID+GmixϕEX.

The first two terms on the r.h.s. of Equation (21) are defined as follows:

(22) GmixϕREF=∑i=1n xiϕ Giϕ0,

(23) GmixϕID=−RT∑i=1nxiϕln⁡xiϕ,

where n is the number of components and is equal to 2 in this work. The molar excess Gibbs energy GmixϕEX in the case of solid and liquid solutions is given by the Redlich–Kister polynomial equation:

(24) GmixϕEX=∑i=1n∑j=i+1nxiϕxjϕ∑k=0lϕLi,j:Vaϕkxiϕ−xjϕk.

The temperature dependence of the interaction parameters Li,j:Vaϕk are expressed as

(25) Li,j:Vaϕk=ai,j:Vaϕk+bi,j:VaϕkT.

The gas phase solution was described as ideal such that GmixgasID=0.

### 3.3. Parameters Estimation and Optimization Procedures

A detailed description of the stepwise procedures followed for estimating and optimizing model parameters is reported in this section, and the values obtained are summarized in Table 1 and Table 2. Elemental Nb and Zr parameters were fitted using non-linear regression techniques through custom Fortran codes and leveraging IMSL numerical libraries. Pandat Software Education 2024 [54] was utilized to compute the phase diagram and optimize the solution phase interaction parameters by implementing model equations in a manually composed TDB file, available as Supplementary Material in the online version of this work. As thoroughly explained in the literature review section, heat capacity, enthalpy data, and melting and boiling points were used to optimize the thermodynamic parameters. As a general guideline, the number of fitted parameters are reduced as much as possible, to possibly avoid over-parametrization, particularly when experimental data are limited. Accordingly, when independent parameter estimation methods are available, they were preferentially adopted.

The standard reference stable phase (α) of Nb and Zr are BCC A2 and HCP A3, respectively. Einstein temperature of these phases was estimated proportionally to the low temperature limit for the Debye temperature $\theta_{D}(-3)$ by using the relation $\theta_{E}^{\alpha }≅0.717\theta_{D}^{\alpha }(-3)$ [55] with $\theta_{D,Nb}^{\alpha }(-3)$ = 276 K and $\theta_{D,Zr}^{\alpha }(-3)$ = 290 K [56,57]. Parameters ν, a, and b for both the α phases mentioned above were then calculated by fitting using the heat capacity data reported in the literature [30,38]. The best fitting outcome was obtained for ν=4 ($R_{adj}^{2}=0.99947$) in the case of BCC A2 Nb, while ν equal to 2 ($R_{adj}^{2}=0.99972$) was found to be the best choice for HCP A3 Zr.

The hexagonal crystal structure of Zr is the stable phase from 0 K to 1139 K; then, a phase transition to the BCC A2 structure occurs. To the best of the authors’ knowledge, the Debye temperature of the last phase is not available in the literature, and so the Einstein temperature of the last phase cannot be estimated as reported above for the HCP A3 phase. Therefore, $\theta_{E,Zr}^{BCC\_A2}$ was obtained by fitting together with the other parameters ν, a, and b. Heat capacity experimental data are used for fitting by also imposing the constraint GZrBCC A20=GZrHCP A30 at the transition temperature. The difference $E_{0,Zr}^{BCC\;A2}-E_{0,Zr}^{HCP\;A3}$, which is necessary to calculate GZrBCC A20 (cf. Equation (6)) is taken from the Open Quantum Materials Database (OQMD) [58,59], and it is reported in Table 1. Best fitting results were obtained for ν=4 ($R_{adj}^{2}=1$). It is worth noting that, for the BCC A2 phase, the best fitting results have been obtained with the same value of ν for both Nb and Zr.

**Table 1:** Thermodynamic model parameters of elemental Nb and Zr.

Element	Phase ϕ	$E_{0}^{\phi }-E_{0}^{\alpha }$ (J mol^−1^)	${∆S}^{\alpha \to \phi }$ (J mol^−1^ K^−1^)	$\theta_{E}^{\phi }$ (K)	$\nu^{\phi }$ (-)	$a^{\phi }$ (J mol^−1^ K^−2^)	$b^{\phi }$ (J mol^−1^ K^-ν−1^)
Nb	FCC_A1	31,070 ^(a)^	−1.7 ^(b)^	211.85	4	2.9658 × 10^−3^	1.9255 × 10^−13^
	BCC_A2 (*α*)	0	-	197.89	4	2.9658 × 10^−3^	1.9255 × 10^−13^
	HCP_A3	28,658 ^(a)^	−2.4 ^(b)^	217.88	4	2.9658 × 10^−3^	1.9255 × 10^−13^
	amorphous	17,568	-	197.88	-	4.6 × 10^−3 (c)^	-
Zr	FCC_A1	3860 ^(a)^	0.9 ^(b)^	200.56	4	7.3793 × 10^−3^	4.8187 × 10^−7^
	BCC_A2	7719 ^(a)^	-	140.79	4	2.3062 × 10^−3^	3.0624 × 10^−13^
	HCP_A3 (*α*)	0	-	207.93	2	7.3793 × 10^−3^	4.8187 × 10^−7^
	amorphous	11,840	-	140.79	-	1.65 × 10^−3 (c),(d)^	-
Element	Phase ϕ	$A^{liq}$ (J mol^−1^)		$B^{liq}$ (J mol^−1^ K^−1^)		$C^{liq}$ (J mol^−1^ K)	
Nb	liquid	29,076		8.3144		−1.0000 × 10^−8^	
Zr	liquid	48,452		8.3144		−9.6122 × 10^−1^	
Element	Phase ϕ	Parameter	Value	Element	Phase ϕ	Parameter	Value
Nb	gas	$a_{0}^{gas}$	7.1937 × 10^5^	Zr	gas	$a_{0}^{gas}$	5.9956 × 10^5^
		$a_{1}^{gas}$	−3.3777 × 10^1^			$a_{1}^{gas}$	−7.9467 × 10^0^
		$a_{2}^{gas}$	3.6289 × 10^1^			$a_{2}^{gas}$	−1.0028 × 10^2^
		$a_{3}^{gas}$	6.4223 × 10^−3^			$a_{3}^{gas}$	−6.6663 × 10^−2^
		$a_{4}^{gas}$	−9.3483 × 10^−7^			$a_{4}^{gas}$	5.6647 × 10^−5^
		$a_{5}^{gas}$	5.8594 × 10^−11^			$a_{5}^{gas}$	−3.5606 × 10^−8^
		$a_{6}^{gas}$	−1.1731 × 10^−15^			$a_{6}^{gas}$	1.5301 × 10^−11^
		$a_{7}^{gas}$	0			$a_{7}^{gas}$	−4.4752 × 10^−15^
		$a_{8}^{gas}$	0			$a_{8}^{gas}$	8.7741 × 10^−19^
		$a_{10}^{gas}$	0			$a_{10}^{gas}$	−1.1042 × 10^−22^
		$a_{11}^{gas}$	0			$a_{11}^{gas}$	8.0661 × 10^−27^
		$a_{12}^{gas}$	0			$a_{12}^{gas}$	−2.6006 × 10^−31^

^(a)^ [58,59]; ^(b)^ [46]; ^(c)^ [60]; ^(d)^ [38].

The procedures described above cannot be applied for FCC A1 Nb and Zr, and in the case of HCP A3 Nb. In fact, when dealing with phases that are not experimentally accessible at any temperature, experimental data are not available, and consequently, fitting is not applicable. This implies the problem of determining the parameters $\theta_{E}$, a, b, and ν for such phases, which will be referred to as metastable ones in what follows. As far as the Einstein temperature of the latter ones is concerned, a method proposed by Dinsdale and coworkers is adopted in this work [24]. Specifically, the difference in molar entropy between a metastable phase ϕ and the reference stable phase α, ${∆S}^{\alpha \to \phi }$, is expressed as a function of the ratio between the Einstein temperatures of the two phases:

(26) $${∆S}^{\alpha \to \phi }=3R\ln\left(\frac{\theta_{E}^{\phi }}{\theta_{E}^{\alpha }}\right).$$

Therefore, if ${∆S}^{\alpha \to \phi }$ is known, the Einstein temperature of the metastable phase can be calculated by using the following relation:

(27) $$\theta_{E}^{\phi }=\theta_{E}^{\alpha }\exp\left(-\frac{{∆S}^{\alpha \to \phi }}{3R}\right).$$

The values of ${∆S}^{\alpha \to \phi }$ used in this work are taken from the literature [46] and reported in Table 1, along with the calculated Einstein temperatures. Regarding the remaining parameters of the metastable phases, and according to the current approach adopted by the CALPHAD community, their values are assigned equal to the corresponding reference stable phase α (see Table 1).

Thermodynamic properties of liquid Nb and Zr are assessed using the two-state model for liquid–amorphous phases presented in Section 3.1. The number of adjustable parameters is reduced as much as possible due to the narrow temperature range that characterized the experimental data available for the liquid phases. Specifically, coefficient $a^{am}$ was set equal to the value of the electronic heat capacity of Nb at high temperature, since it is assumed that the electronic capacity of liquid–amorphous phase is close to that of solid at the melting point [61,62]. However, based on the author’s knowledge, no electronic heat capacity for Zr at high temperatures is reported in the literature. Thus, this value is obtained by multiplying the electronic heat capacity of Zr at low temperatures by the ratio between electronic heat capacity at high and low temperatures calculated for Nb, assuming that this ratio is the same for both Nb and Zr.

Experimental data of liquid heat capacity for both Nb and Zr are limited to the nearby melting point, so that the high-temperature behavior of this property is unknown. Therefore, it was set $b^{am}=0$. Parameter $B^{liq}$ in Equation (12) was fixed to the communal entropy R [63], while $A^{liq}$ and $C^{liq}$ were optimized along with cohesive energy difference $E_{0}^{am}-E_{0}^{\alpha }$. In this regard, it should be noted that since $\left(\xi_{eq}-\xi_{eq}^{2}\right)>0$ in Equation (14), C must be negative to maintain the derivative positive as T→∞. Heat capacity and enthalpy data were used for parameters optimization by also imposing for both metals the condition Gliq−am0=GBCC_A20 at the melting temperature. In the case of liquid Zr, heat data capacity from Paradis et al. [39] was used after being corrected to account for the more accurate emissivity estimation reported by Sakata et al. [40].

Lastly, coefficients $a_{i}^{gas}$ appearing in Equation (20) and reported in Table 1 were obtained for both Nb and Zr by fitting literature data [30,38], while interaction parameters used for the thermodynamic description of Nb-Zr binary solid and liquid solution phases are reported in Table 2.

**Table 2:** Interaction parameters of binary phase solutions modeled in this work. Note that all the values are given as SI unit J/mol formula unit.

BCC A2: ${\left(Nb,Zr\right)}_{1}{\left(Va\right)}_{3}$	
LNb,Zr:VaBCC A20=15911+3.5 T	[44]
LNb,Zr:VaBCC A21=3919−1.091 T	[44]
HCP A3: ${\left(Nb,Zr\right)}_{1}{\left(Va\right)}_{0.5}$	
LNb,Zr:VaHCP A30=24,411	[44]
Liquid:${\left(Nb,Zr\right)}_{1}$	
LNb,Zrliq0=10,311	[44]
LNb,Zrliq0=10,296.1	This work
LNb,Zrliq1=6709	[44]
LNb,Zrliq1=4286.5	This work

## 4. Results and Discussion

In what follows, the thermodynamic properties of elemental Nb and Zr and the associated Nb-Zr binary phase diagram calculated using the procedure described in Section 3 are compared with both experimental data and previous modeling results.

**Figure 1:**
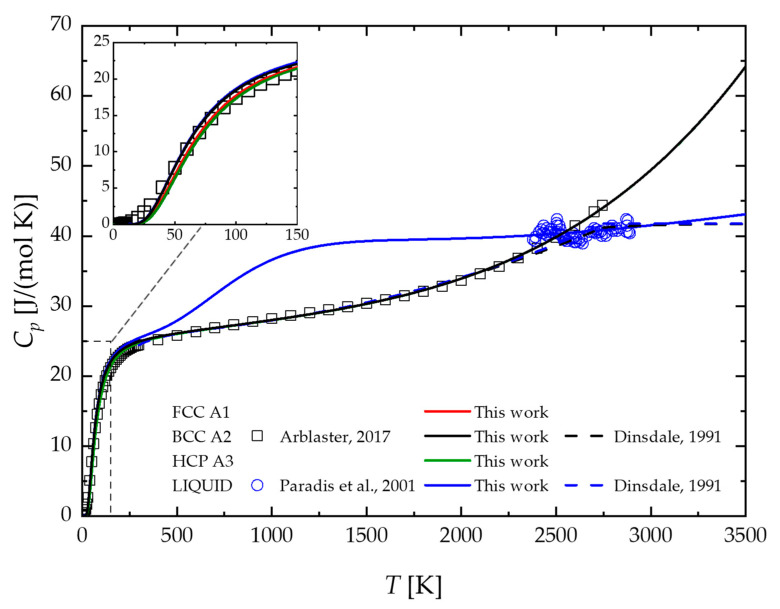
Temperature profiles of Nb heat capacity in comparison with experimental data. The color code should read as follows: FCC A1 (red), BCC A2 (black), HCP A3 (green), and liquid (blue). Arblaster, 2017 [30]; Paradis et al., 2001 [35]; Dinsdale, 1991 [46].

The calculated heat capacities of FCC A1, BCC A2, HCP A3, and liquid Nb are shown in Figure 1. These results are compared with those calculated using the thermodynamic description previously reported by Dinsdale [46] and the experimental data used for optimization. An excellent agreement with the empirical values can be observed for the BCC phase up to the melting point. On the contrary, Figure 1 indicates that the Nb BCC description by Dinsdale [46] underestimates the heat capacity of this phase near the melting temperature (2745 K). The better agreement shown in this work may be due to the more recent experimental data provided by Arblaster [30], which are considered in this work for optimization. On the contrary, no experimental data are available for the metastable FCC and HCP Nb phases. Accordingly, the differences compared to the BCC phase are confined to the low-temperature range (see inset in Figure 1), while at higher temperatures, the heat capacity of FCC and HCP Nb results are very similar to those of the BCC phase. This behavior is not surprising since the heat capacity coefficients a and b of FCC and HCP were imposed equal to the BCC ones.

Figure 1 shows a good agreement between the calculated heat capacity of the liquid Nb phase and the corresponding experimental data. However, the latter ones are significantly scattered and available for a limited temperature range due to the well-known experimental difficulties in measuring this property. A significant difference compared to the previous 2nd generation CALPHAD description provided by Dinsdale [46] can be observed below the melting point. Above this temperature, the deviation is smaller, even if the typical assumption of constant heat capacity for the liquid phase previously adopted clearly appears as just an approximation.

**Figure 2:**
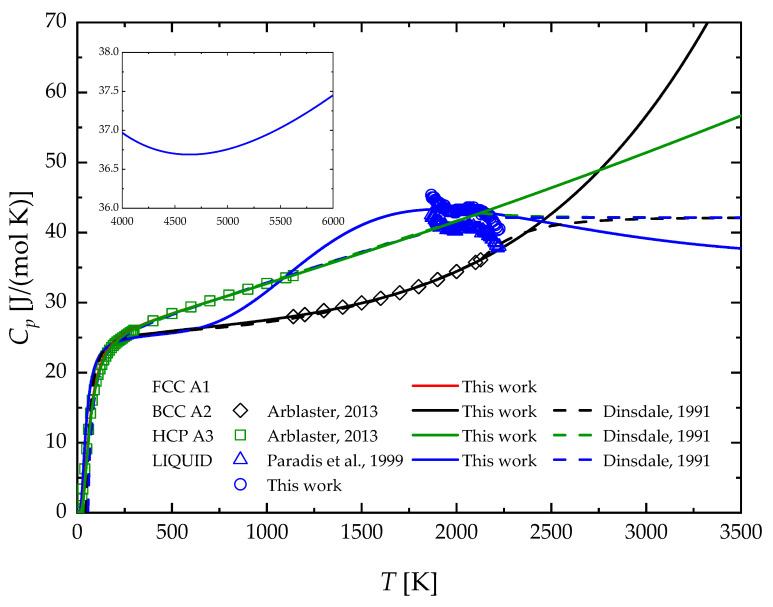
Temperature profiles of Zr heat capacity in comparison with experimental data. The color code should read as follows: FCC A1 (red), BCC A2 (black), HCP A3 (green), and liquid (blue). Arblaster, 2013 [38]; Paradis et al., 1999 [39]; Dinsdale, 1991 [46].

The same analysis for the case of Zr can be performed by referring to Figure 2, where the calculated heat capacity of the corresponding solid (BCC, FCC, HCP) and liquid phases are shown. Available experimental data and previous second generation descriptions are also reported for the sake of comparison. A very good agreement is obtained between the calculated heat capacity of HCP and BCC Zr and the related experimental data. Regarding the metastable FCC phase, no empirical values are available, and, therefore, its calculated heat capacity is very similar to that of the stable room temperature phase, as in the case of Nb. Compared to the case of Nb shown in Figure 1, the heat capacity of liquid Zr displays a pronounced maximum at about T = 1900 K, while a flat minimum can be observed at higher temperatures (see inset in Figure 2). The comparison of the calculated heat capacity of the liquid phase with the experimental data is less satisfactory than that of Nb. This can be ascribed to the observed peculiar behavior of these properties as a function of the temperature.

**Figure 3:**
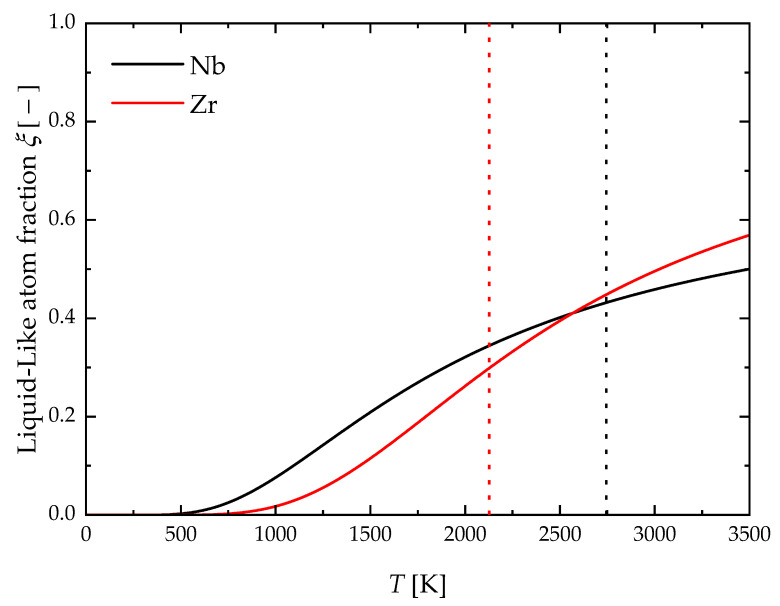
Temperature profiles of equilibrium liquid-like atom fraction $\xi_{eq}$. Melting temperatures are indicated as straight vertical dotted lines ($T_{m}^{Nb}=2745\;K$ and $T_{m}^{Zr}=2127\;K$).

Figure 3 shows the calculated temperature profiles of the liquid-like atoms fraction, $\xi_{eq}$, in liquid Nb and Zr using Equations (11) and (13). Both profiles exhibit the typical sigmoid shape reported by previous authors [23,53,64,65]. It is seen that the fraction of liquid-like atoms increases on heating, and, for Nb, it reaches around 43% at the melting temperature of 2745 K, while a value of approximately 30% is approached for Zr at the melting point of 2127 K. These results are consistent with those reported for W (about 40%) at the melting temperature of 3687 K [23,65].

**Figure 4:**
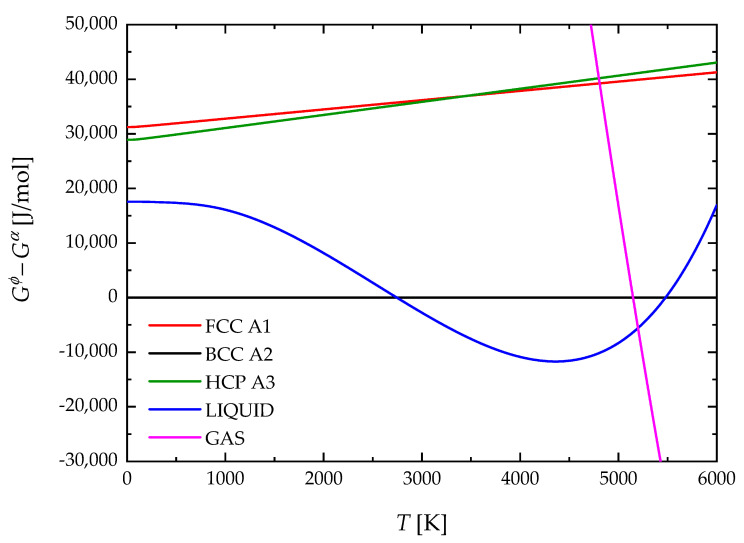
Molar Gibbs free energy of phases of Nb relative to BCC A2.

**Figure 5:**
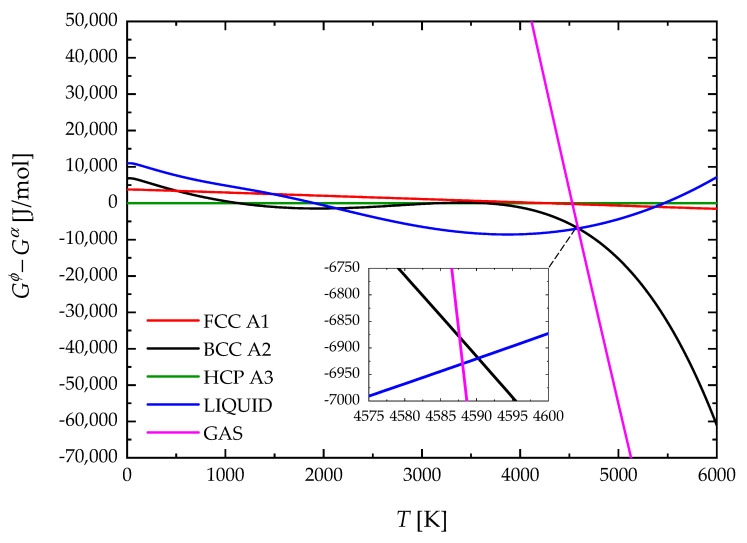
Molar Gibbs free energy of phases of Zr relative to HCP A3.

The molar Gibbs free energy, Gϕ0, of all phases for Nb are reported in Figure 4. It can be observed that the BCC phase would re-stabilize at a high temperature (T = 5500 K) if the gas phase is suppressed. A similar behavior is shown in Figure 5 for the case of Zr. Here, the re-stabilization of the BCC phase takes place at T = 4590 K if the gas phase is not considered. Stability at high temperatures of solid phases at the expense of the liquid phase was already reported by Dinsdale [46]. However, if the gas phase is considered, Figure 4 and Figure 5 show that it becomes stable before the solid phase can re-stabilize. Figure 4 indicates that the intersection of liquid and gas phase G curves for Nb occurs at 5199 K. This value is in good agreement with the normal boiling point (NBP) of Nb reported by Arblaster [30], i.e., 5197.42 K. Similarly, for the case of Zr, Figure 5 shows that the intersection of liquid and gas phase G curves takes place at 4588 K. The latter value also agrees with the NBP of Zr reported by Arblaster [38] (4636.07 K). It is worth pointing out that, unlike previous third-generation CALPHAD modeling approaches that implement the equal entropy criterion (EEC) [66], the addition of an ideal gas phase was alternatively considered in the present work to avoid the re-stabilization of solid phases at high temperatures.

**Figure 6:**
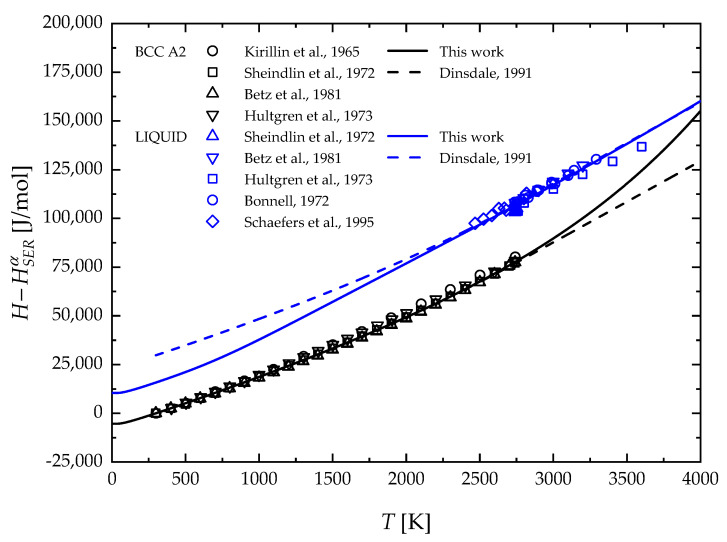
Temperature profiles of molar enthalpy of solid BCC A2 and liquid Nb in comparison with experimental data. The color code should read as follows: BCC A2 (black) and liquid (blue). Kirillin et al., 1965 [31]; Sheindlin et al., 1972 [32]; Betz et al., 1981 [33]; Hultgren et al., [34]; Bonnell, 1972 [36]; Schaefers et al., 1995 [37]; Dinsdale, 1991 [46].

Figure 6 shows a comparison between experimental data and model results in terms of the molar enthalpy of liquid and solid BCC Nb. The blue solid curve should be interpreted as fitting since liquid phase enthalpy data were used to determine model parameters (see Section 3). Vice versa, the solid black curve represents the model prediction, which agrees very well with the corresponding experimental data. Previous calculations of Dinsdale [46] are also reported in Figure 6 for the sake of comparison. It can be clearly observed that there is a close agreement in the temperature ranges where experimental data are available. On the contrary, a significant difference between the two model results appears in the metastable ranges, i.e., below and above the melting temperature for the liquid and solid BCC phases, respectively.

**Figure 7:**
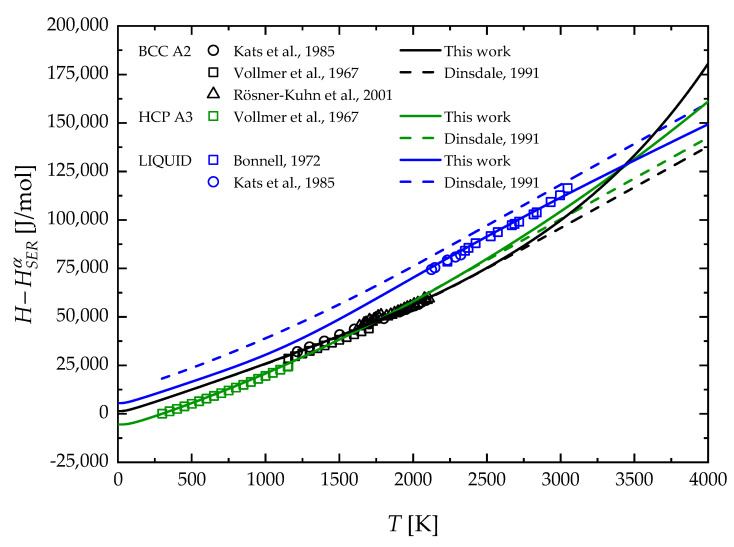
Temperature profiles of molar enthalpy of solid HCP A3, BCC A2, and liquid Zr in comparison with experimental data. The color code should read as follows: BCC A2 (black), HCP A3 (green), and liquid (blue). Bonnell, 1972 [36]; Kats et al., 1985 [41]; Vollmer et al., 1967 [42]; Rösner-Kuhn et al., 2001 [43]; Dinsdale, 1991 [46].

Similar considerations can be made for Zr, whose corresponding temperature profiles of molar enthalpy are plotted in Figure 7. Also, in this case, the solid blue curve should be read as a fitting result, while the solid black and green lines represent the predicted molar enthalpy of BCC and HCP phases, respectively. Both fitting and modeling outcomes match the corresponding experimental data very well. In contrast, significant differences emerge when compared to the second CALPHAD generation [46]. In the case of the solid phases, molar enthalpy estimated in this work shows higher values for temperatures exceeding about 2500 K, with expanding differences as the temperature increases. Moreover, the present calculation results in lower values for the molar enthalpy of the liquid phase in the entire investigated temperature range, with a better agreement with the available experimental data.

**Figure 8:**
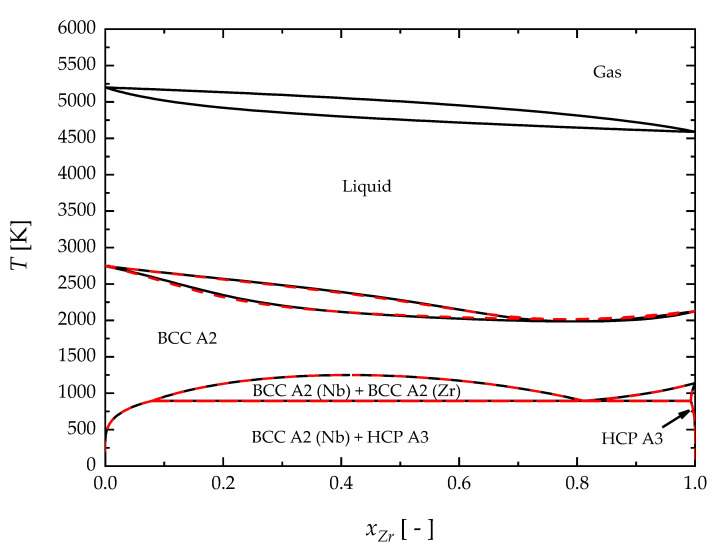
The Nb-Zr phase diagram calculated using the present thermodynamic description (black line) and the parameters proposed by [44] (dashed red line).

The calculated phase diagram of the Nb-Zr system is presented in Figure 8, and the comparison with the previous assessment proposed by Fernández Guillermet (1991) is also shown [44]. An acceptable agreement is observed between the two modeling results for the BCC/liquid equilibrium region, while a perfect superposition can be seen for the solid–solid equilibria at lower temperatures. The liquid–gas equilibrium is also shown to confirm that artificial re-stabilization of the Nb-Zr BCC solid solution phase at high temperatures does not occur, as already reported in Figure 4 and Figure 5 for the elemental components.

**Figure 9:**
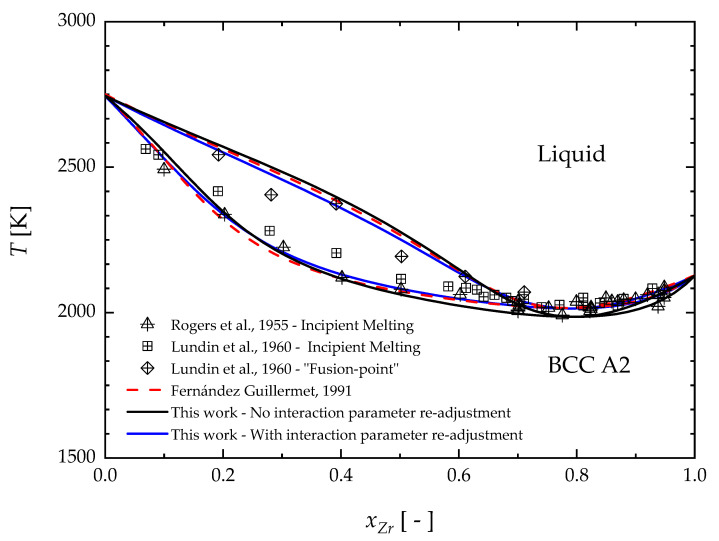
The BCC A2/Liquid equilibrium boundaries of the Nb-Zr phase diagram calculated using the present thermodynamic description (solid black and blue lines) and the parameters proposed by Fernández Guillermet, 1991 [44] (dashed red lines), compared with experimental data. Rogers et al., 1955 [67]; Lundin et al., 1960 [68].

The comparison between the calculated phase diagram and experimental data for the BCC/liquid equilibrium region is presented in Figure 9. Specifically, black lines represent the BCC/liquid equilibrium obtained in this work by using the proposed description for elemental Nb and Zr, while keeping the same interaction parameters previously provided by Fernández Guillermet (1991) [44]. Deviation of the black lines with respect to the dashed red curves can be thus seen as the effect of the new third-generation description. This finding is somehow expected since the mixing thermodynamic quantities are evaluated by taking the pure components as a reference state. In order to have a better agreement with the experimental data, the interaction parameters for the liquid solution were re-adjusted in this work. In Figure 9, the blue lines represent the newly assessed solid–liquid phase boundaries. New values of these interaction parameters are reported in Table 2. Their temperature dependence has been neglected according to the previous assessment [44]. On the contrary, the interaction parameters of BCC and HCP solid solution were retained from Fernández Guillermet’s assessment, owing to the good agreement between the present description of the solid elements and the previous one.

**Figure 10:**
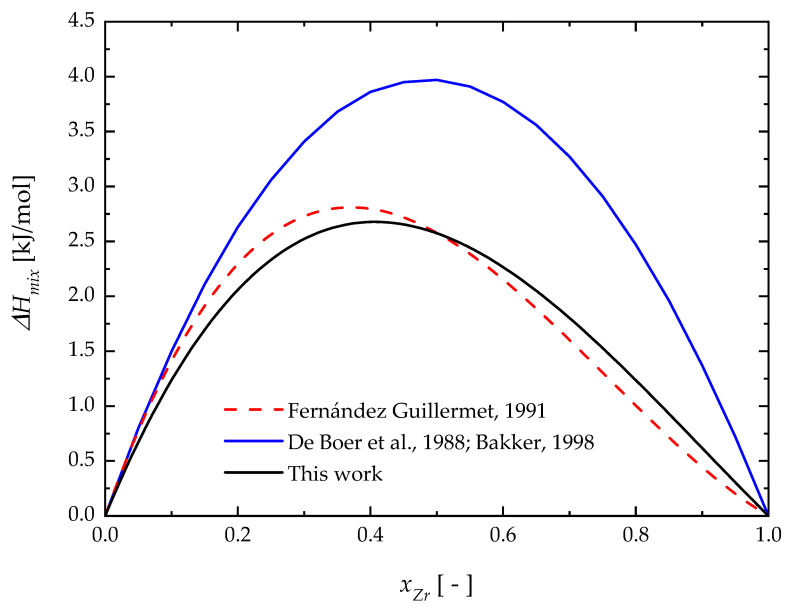
The enthalpy of mixing ($∆H_{mix}$) of liquid Nb-Zr alloys. Fernández Guillermet, 1991 [44]; De Boer et al., 1988 [69]; Bakker, 1998 [70].

To evaluate the reliability of the adjusted liquid interaction parameters, the resulting enthalpy of mixing (${∆H}_{mix}$) is compared in Figure 10 to that obtained by Fernández Guillermet [44]. It can be seen that ${∆H}_{mix}$ calculated in this work is different than the previous estimation. Unfortunately, no experimental data are available to properly compare the calculated results. For the sake of completeness, the enthalpy of mixing provided by the well-known Miedema’s model [69,70] is also shown in Figure 10. However, only a qualitative agreement is observed between the present estimation and the Miedema model. The latter displays higher values of ${∆H}_{mix}$ and a higher symmetry with respect to the alloy composition. Compared to Fernández Guillermet’s results [44], present calculations give a lower maximum shift to Zr-richer alloys.

**Figure 11:**
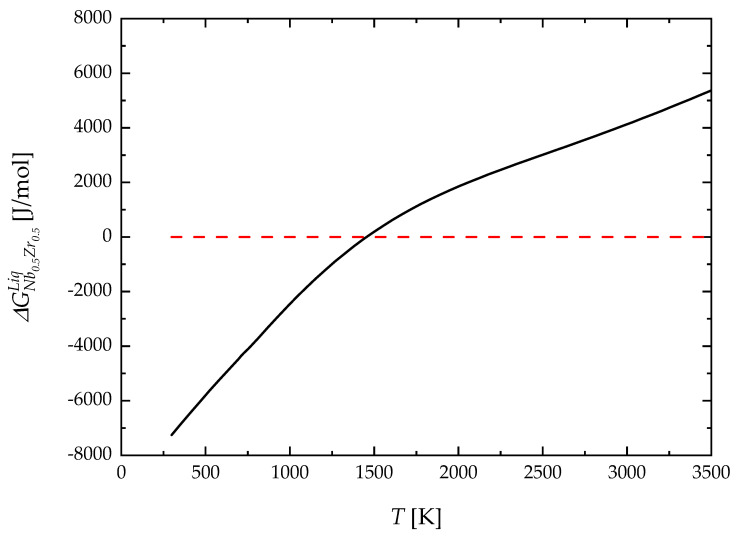
Molar Gibbs free energy of liquid $Nb_{0.5}{Zr}_{0.5}$ solution.

With the aim to highlight the effect of the new description of elemental Nb and Zr on the relative thermodynamic stability of the liquid phase, the difference between the molar Gibbs free energy of an equimolar Nb-Zr liquid solution calculated with the parameters obtained in this work and the one obtained by using the previous assessment provided by [44], ${∆G}_{{Nb}_{0.5}{Zr}_{0.5}}^{liq}$, is plotted in Figure 11 as a function of temperature. It can be seen that the description based on the 3rd generation approach significantly alters the thermodynamic stability of this liquid solution. Specifically, a higher relative stability (i.e., lower value of the Gibbs free energy) is shown up to T = 1440 K, while a decreasing relative stability can be observed for higher temperatures. It is then apparent that the approach proposed in this work can potentially model metallic liquids more accurately across a wider range of conditions, including those where metastable or undercooled states play a significant role.

## 5. Conclusions

In this work, the third-generation CALPHAD approach is applied to achieve a more refined thermodynamic description of elemental Nb and Zr, with a particular emphasis on their liquid phases. It allowed for a significant improvement in the representation of their liquid thermodynamic properties, particularly the peculiar heat capacity behavior near the melting point. A new treatment for solid unary endmembers is suggested with a single Gibbs energy expression, which is used for each phase in the entire temperature range. It is shown that if the gas phase is included in the system description, this treatment does not require the equal-entropy criterion (EEC), and no re-stabilization of solids occurs at high temperatures. The Gibbs energy expression of ideal amorphous phases is revised and added to the descriptions of liquid Nb and liquid Zr. The prediction of normal boiling points for both Nb and Zr is in good agreement with those reported in the literature. By applying the new unary models of Nb and Nb, the Nb-Zr system was assessed for the third generation of thermodynamic descriptions. Overall, a good fit to the experimental data was obtained. A precise optimization procedure is suggested in this work to estimate all the adjustable parameters.

The thermodynamic approach proposed in this work for refractory metals and their alloys can provide useful insights for their practical exploitation. Indeed, the improved description of liquid Nb and Zr and the resulting accuracy in phase stability predictions are essential for optimizing materials processing in high-performance applications, such as nuclear reactors and aerospace components, where precise control over the liquid phase behavior is mandatory. The application of the same approach to describe the thermodynamic properties of hafnium is along the way.

## Data Availability

The original contributions presented in the study are included in the article and Supplementary Material, further inquiries can be directed to the corresponding author.

## Supplementary Materials

The following supporting information can be downloaded at: https://www.mdpi.com/article/10.3390/ma17235978/s1, Pandat input file.
