# Supplementary material for: Third-Generation CALPHAD Modeling of Elemental Nb and Zr and Partial Re-Assessment of Their Binary Phase Diagram
Source: Materials (Basel). 2024 Dec 6;17(23):5978. doi: 10.3390/ma17235978 (PMC11643692; doi:10.3390/ma17235978)
Supplement: Supplementary file 1 [file materials-17-05978-s001.zip › materials-3332459-supplementary.pdf]

```
$=================================================================
$       Nb-Zr
$
$       Traversari 2024
$=================================================================
ELEMENT VA   VACUUM                   0.0000E+00  0.0000E+00  0.0000E+00!
ELEMENT NB   BCC_A2                   9.2906E+01  5.2200E+03  3.6270E+01!
ELEMENT ZR   HCP_A3                   9.1224E+01  5.5663E+03  3.9181E+01!
$=================================================================
$ Pure Nb
$-----------------------------------------------------------------
$ SER BCC_A2
$-----------------------------------------------------------------
 FUNCTION ANBS 0.01 -197.892*T**(-1); 6000 N !
 FUNCTION BNBS 0.01 EXP(ANBS)-1; 6000 N !
 FUNCTION CNBS 0.01 LN(-BNBS); 6000 N !
 FUNCTION GEINNB1 0.01 3*8.31446261815324*CNBS*T; 6000 N !
$
 FUNCTION GEINNBS 0.01 1.5*8.31446261815324*197.892+GEINNB1; 6000 N !
$
 FUNCTION GHSERNB 0.01
  -7839.8207231
  -(2.9658403561982692E-3/2)*T**2
  -(1.9254759017419896E-13/20)*T**5+GEINNBS;
 6000 N !
$-----------------------------------------------------------------
$ LIQUID
$-----------------------------------------------------------------
 FUNCTION ANBL 0.01 -197.87819982209612*T**(-1); 6000 N !
 FUNCTION BNBL 0.01 EXP(ANBL)-1; 6000 N !
 FUNCTION CNBL 0.01 LN(-BNBL); 6000 N !
 FUNCTION GEINNB2 0.01 3*8.31446261815324*CNBL*T; 6000 N !
$
 FUNCTION GEINNBL 0.01
    1.5*8.31446261815324*197.87819982209612+GEINNB2; 6000 N !
$
 FUNCTION A2STNBL 0.01 29075.686755071936
                       -8.31446261815324*T
                       -9.9999987089888491E-9*T*LN(T); 6000 N !
$
 FUNCTION B2STNBL 0.01 -A2STNBL*T**(-1)*8.31446261815324**(-1); 6000 N !
$
 FUNCTION C2STNBL 0.01 1+EXP(B2STNBL); 6000 N !
$
 FUNCTION D2STNBL 0.01 LN(C2STNBL); 6000 N !
$
 FUNCTION G2STNBL 0.01 -8.31446261815324*T*D2STNBL; 6000 N !
$
 FUNCTION GLIQNB 0.01
  17567.865435378695-7839.8207231
  -(0/3)*T
  -(4.6e-3/2)*T**2
  +GEINNBL+G2STNBL;
 6000 N !
```

```
$--------------------------------------------------------------------
$ HCP_A3
$--------------------------------------------------------------------
 FUNCTION ANBS2 0.01 -217.878879849845*T**(-1); 6000 N !
 FUNCTION BNBS2 0.01 EXP(ANBS2)-1; 6000 N !
 FUNCTION CNBS2 0.01 LN(-BNBS2); 6000 N !
 FUNCTION GEINNB12 0.01 3*8.31446261815324*CNBS2*T; 6000 N !
$
 FUNCTION GEINNBS2 0.01 1.5*8.31446261815324*217.878879849845+GEINNB12; 6000 N !
$
 FUNCTION GHCPNB 0.01
  +28658-7839.8207231
  -(2.9658403561982692E-3/2)*T**2
  -(1.9254759017419896E-13/20)*T**5+GEINNBS2;
 6000 N !
$--------------------------------------------------------------------
$ FCC_A1
$--------------------------------------------------------------------
 FUNCTION ANBS3 0.01 -211.849424782754*T**(-1); 6000 N !
 FUNCTION BNBS3 0.01 EXP(ANBS3)-1; 6000 N !
 FUNCTION CNBS3 0.01 LN(-BNBS3); 6000 N !
 FUNCTION GEINNB13 0.01 3*8.31446261815324*CNBS3*T; 6000 N !
$
 FUNCTION GEINNBS3 0.01 1.5*8.31446261815324*211.849424782754+GEINNB13; 6000 N !
$
 FUNCTION GFCCNB 0.01
  +31070-7839.8207231
  -(2.9658403561982692E-3/2)*T**2
  -(1.9254759017419896E-13/20)*T**5+GEINNBS3;
 6000 N !
$--------------------------------------------------------------------
$ GAS
$--------------------------------------------------------------------
 FUNCTION GGASNB 298.15
  +719366.56677349925+36.288824191427899*T
  -(33.776784410158044)*T*LN(T)
  -(-1.2844506901083384E-002/2)*T^2
  -(5.6090019538180871E-006/6)*T^3
  -(-7.0313199956392005E-010/12)*T^4
  -(2.3461778601403567E-014/20)*T^5;
 6000.00 N !
$====================================================================
$ Pure Zr
$--------------------------------------------------------------------
$ SER HCP A3
$--------------------------------------------------------------------
 FUNCTION AZRS 0.01 -207.93*T**(-1); 6000 N !
 FUNCTION BZRS 0.01 EXP(AZRS)-1; 6000 N !
 FUNCTION CZRS 0.01 LN(-BZRS); 6000 N !
 FUNCTION GEINZR1 0.01 3*8.31446261815324*CZRS*T; 6000 N !
$
 FUNCTION GEINZRS 0.01 1.5*8.31446261815324*207.93+GEINZR1; 6000 N !
$
 FUNCTION GHSERZR 0.01
```

```
  -8068.12096481
  -(7.3793332872920296E-3/2)*T**2
  -(4.8186614182508526E-7/6)*T**3+GEINZRS;
 6000 N !
$------------------------------------------------------------------
$ BCC A2
$------------------------------------------------------------------
 FUNCTION AZRB 0.01 -140.79318243245876*T**(-1); 6000 N !
 FUNCTION BZRB 0.01 EXP(AZRB)-1; 6000 N !
 FUNCTION CZRB 0.01 LN(-BZRB); 6000 N !
 FUNCTION GEINZR2 0.01 3*8.31446261815324*CZRB*T; 6000 N !
$
 FUNCTION GEINZRB 0.01 1.5*8.31446261815324*140.79318243245876+GEINZR2; 6000 N !
$
 FUNCTION GBCCZR 0.01
  7718.84278628544-8068.12096481
  -(2.3062286373495460E-003/2)*T**2
  -(3.0623572621929292E-013/20)*T**5+GEINZRB;
 6000 N !
$------------------------------------------------------------------
$ LIQUID
$------------------------------------------------------------------
 FUNCTION AZRL 0.01 -140.79199288701724*T**(-1); 6000 N !
 FUNCTION BZRL 0.01 EXP(AZRL)-1; 6000 N !
 FUNCTION CZRL 0.01 LN(-BZRL); 6000 N !
 FUNCTION GEINZR3 0.01 3*8.31446261815324*CZRL*T; 6000 N !
$
 FUNCTION GEINZRL 0.01
        1.5*8.31446261815324*140.79199288701724+GEINZR3; 6000 N !
$
 FUNCTION A2STZRL 0.01 48452.131253896354
                       -8.31446261815324*T
                       -0.96122022443629274*T*LN(T); 6000 N !
$
 FUNCTION B2STZRL 0.01 -A2STZRL*T**(-1)*8.31446261815324**(-1); 6000 N !
$
 FUNCTION C2STZRL 0.01 1+EXP(B2STZRL); 6000 N !
$
 FUNCTION D2STZRL 0.01 LN(C2STZRL); 6000 N !
$
 FUNCTION G2STZRL 0.01 -8.31446261815324*T*D2STZRL; 6000 N !
$
 FUNCTION GLIQZR 0.01
  11840.414353299520-8068.12096481
  -(0/3)*T
  -(1.65e-3/2)*T**2
  +GEINZRL+G2STZRL;
 6000 N !
$------------------------------------------------------------------
$ FCC_A1
$------------------------------------------------------------------
 FUNCTION AZRS2 0.01 -200.561268392612*T**(-1); 6000 N !
 FUNCTION BZRS2 0.01 EXP(AZRS2)-1; 6000 N !
 FUNCTION CZRS2 0.01 LN(-BZRS2); 6000 N !
```

```
 FUNCTION GEINZR12 0.01 3*8.31446261815324*CZRS2*T; 6000 N !
$
 FUNCTION GEINZRS2 0.01 1.5*8.31446261815324*200.561268392612+GEINZR12; 6000 N !
$
 FUNCTION GFCCZR 0.01
  3860-8068.12096481
  -(7.3793332872920296E-3/2)*T**2
  -(4.8186614182508526E-7/6)*T**3+GEINZRS2;
 6000 N !
$-----------------------------------------------------------------
$ GAS
$-----------------------------------------------------------------
 FUNCTION GGASZR 298.15
  +599556.64189519919-100.27782991831782*T
  -(7.9466833991196992)*T*LN(T)
  -(0.13325133447396659/2)*T^2
  -(-3.3987957113500460E-004/6)*T^3
  -(4.2726638782769705E-007/12)*T^4
  -(-3.0600964185113789E-010/20)*T^5
  -(1.3425689112824461E-013/30)*T^6
  -(-3.6851112216224706E-017/42)*T^7
  -(6.1837151695107752E-021/56)*T^8
  -(-5.8076210733788838E-025/72)*T^9
  -(2.3405508198679943E-029/90)*T^10;
 6000.00 N !
$--------------------------------------------------------------------------------
 TYPE_DEFINITION % SEQ *!
 DEFINE_SYSTEM_DEFAULT ELEMENT 2 !
 DEFAULT_COMMAND DEF_SYS_ELEMENT VA /- !
$--------------------------------------------------------------------------------
 PHASE LIQUID %  1  1.0  !
   CONSTITUENT LIQUID :NB,ZR :  !
   PARAMETER G(LIQUID,NB;0)     298.15  +GLIQNB;             6000 N !
   PARAMETER G(LIQUID,ZR;0)     298.15  +GLIQZR;             6000 N !
$--------------------------------------------------------------------------------
$       GUILLERMET
$--------------------------------------------------------------------------------
$   PARAMETER G(LIQUID,NB,ZR;0)  298.15  +10311;             6000 N !
$   PARAMETER G(LIQUID,NB,ZR;1)  298.15  +6709;              6000 N !
$--------------------------------------------------------------------------------
$       Traversari
$--------------------------------------------------------------------------------
   PARAMETER G(LIQUID,NB,ZR;0)  298.15  +10296.1;             6000 N !
   PARAMETER G(LIQUID,NB,ZR;1)  298.15  +4286.46;              6000 N !
$--------------------------------------------------------------------------------
$   Ready for Optimization
$--------------------------------------------------------------------------------
$   L0, L1 parameter for LIQUID phase
$--------------------------------------------------------------------------------
$   OPTIMIZATION        PAR_1   0       10311;  20000   N !
$   OPTIMIZATION        PAR_2   0       6709;   10000   N !
$   PARAMETER G(LIQUID,NB,ZR;0)  298.15  PAR_1;             6000 N !
$   PARAMETER G(LIQUID,NB,ZR;1)  298.15  PAR_2;              6000 N !
$--------------------------------------------------------------------------------
```

```
$
 PHASE FCC_A1  %  2 1   1 !
   CONSTITUENT FCC_A1  :NB,ZR : VA :  !
   PARAMETER G(FCC_A1,NB:VA;0)     298.15  +GFCCNB;          6000 N !
   PARAMETER G(FCC_A1,ZR:VA;0)     298.15  +GFCCZR;          6000 N !
$
 PHASE BCC_A2  %  2 1   3 !
   CONSTITUENT BCC_A2  :NB,ZR : VA :  !
   PARAMETER G(BCC_A2,NB:VA;0)     298.15  +GHSERNB;          6000 N !
   PARAMETER G(BCC_A2,ZR:VA;0)     298.15  +GBCCZR;          6000 N !
   PARAMETER G(BCC_A2,NB,ZR:VA;0)  298.15  +15911+3.35*T;    6000 N !
   PARAMETER G(BCC_A2,NB,ZR:VA;1)  298.15  +3919-1.091*T;    6000 N !
$
 PHASE HCP_A3  %  2 1   .5 !
   CONSTITUENT HCP_A3  :NB,ZR : VA :  !
   PARAMETER G(HCP_A3,NB:VA;0)     298.15  +GHCPNB;         6000 N !
   PARAMETER G(HCP_A3,ZR:VA;0)     298.15  +GHSERZR;         6000 N !
   PARAMETER G(HCP_A3,NB,ZR:VA;0)  298.15  +24411;          6000 N !
$
 PHASE GASGAS %  1  1.0  !
   CONSTITUENT GASGAS :NB,ZR :  !
   PARAMETER G(GASGAS,NB;0)     298.15  +GGASNB;             6000 N !
   PARAMETER G(GASGAS,ZR;0)     298.15  +GGASZR;             6000 N !
$--------------------------------------------------------------------------------
$NBZR
```
